# Formulation and *In vitro* Interaction of Rhodamine-B Loaded PLGA Nanoparticles with Cardiac Myocytes

**DOI:** 10.3389/fphar.2016.00458

**Published:** 2016-12-06

**Authors:** Antranik Jonderian, Rita Maalouf

**Affiliations:** Department of Sciences, Notre Dame University – LouaizeZouk Mosbeh, Lebanon

**Keywords:** PLGA, nanoparticles, rhodamine B, cardiac myocytes, cytotoxicity

## Abstract

This study aims to characterize rhodamine B (Rh B) loaded poly(D,L-lactide-*co*-glycolide; PLGA) nanoparticles (NPs) and their interactions with cardiac myocytes. PLGA NPs were formulated using single emulsion solvent evaporation technique. The influence of varying parameters such as the stabilizer concentration, the sonication time, and the organic to aqueous ratio were investigated. The diameter, the dispersity, the encapsulation efficiency and the zeta potential of the optimized NPs were about 184 nm, 0.19, 40% and -21.7 mV, respectively. *In vitro* release showed that 29% of the Rh B was released within the first 8 h. Scanning electron microscopy measurements performed on the optimized NPs showed smooth surface and spherical shapes. No significant cytotoxic or apoptotic effects were observed on cardiac myocytes after 24 and 48 h of exposure with concentrations up to 200 μg/mL. The kinetic of the intracellular uptake was confirmed by confocal microscopy and cells took up PLGA NPs within the 1st hours. Interestingly, our data show an increase in the NPs’ uptake with time of exposure. Taken together, we demonstrate for the first time that the designed NPs can be used as potential probes for drug delivery in cardiac myocytes.

## Introduction

The use of nanotechnology in medicine is considered to be a breakthrough in the field of disease treatments. Biodegradable NPs have been used in various medical applications to carry diagnostic imaging agents and to achieve therapeutic drug delivery (Parveen et al., 2012). Due to their versatility and their wide range of properties, they act as potential carriers for the controlled delivery of several types of drugs as they promise to overcome many of the obstacles inherently associated with their administration (Hans and Lowman, 2002; Panyam and Labhasetwar, 2003; Mahapatro and Singh, 2011). The targeted delivery of the encapsulated molecules allows the increase of the concentration of the drug in some parts of the body relative to others, the maintenance of optimum therapeutic drug concentration for sustained periods of time and the reduction of the frequency of dosages taken by the patient. Also, they extend the drug circulation lifetime and reduce side effects (Manoochehri et al., 2013; Cooper and Harirforoosh, 2014; Bandyopadhyay et al., 2015). Various types of NPs have been investigated for drug delivery applications. Polyketal-based NPs were used to deliver drug and RNA silencing, respectively (Aso et al., 2007; Gray et al., 2011; Somasuntharam et al., 2013). On the other side, Xie et al. (2013) developed a carrier based on dextran-co-gelatin NPs to deliver vascular endothelial growth factor for the treatment of peripheral artery disease. Other researchers used micelles, liposomes, as well as gold NPs for drug delivery (Suarez et al., 2015). However, PLGA NPs are most extensively researched owing to their biocompatibility and biodegradability. PLGA degrades by hydrolysis through cleavage of its backbone ester linkages forming biologically compatible and metabolizable moieties (lactic acid and glycolic acid). They can be processed by the body through the citric acid cycle and eliminated as carbon dioxide and water.

Degradation rates from months to years can be attained by tuning relevant parameters such as the molecular weight of PLGA, ratio of lactide to glycolide, drug content and nanoparticle size. PGA is hydrophilic and a highly crystalline polymer with a relatively fast degradation rate (Park, 1995). For instance, when co-polymerized with PLA, it increases its rate of hydration and hydrolysis. Thus, the higher content of PGA leads to a faster degradation with an exception of the 50/50 PLA/PGA blend that exhibits the quicker decomposition rate (Gentile et al., 2014). It has been also reported that high surface area to volume ratio leads to high degradation rate of the matrix (Makadia and Siegel, 2011). Furthermore, an optimal drug release rate from PLGA is very crucial to achieve a sustained delivery. Larger particles have smaller initial burst release and longer sustained release than smaller particles (Mahapatro and Singh, 2011). Moreover, NPs having higher drug content possess a larger initial burst release than those having lower content because of their smaller polymer to drug ratio (Hans and Lowman, 2002).

Despite the extensive literature related to PLGA NPs use, whether described in cardiac tissues or other organ tissues (Yi et al., 2006; Chang et al., 2013; Navarro et al., 2014), yet, to our knowledge, this is the first study where RhB-loaded PLGA NPs were prepared as models to study their kinetic uptake in primary cardiac myocytes *in vitro*. Importantly, PLGA NPs’ cytotoxicity was also studied in comparison to HG toxicity in the same type of cells, mimicking a diabetic milieu. The designed NPs will pave the way for future synthesis of targeted drug-loaded NPs for the treatment of diabetic cardiomyopathy and various heart diseases. Hyperglycemia is a major risk factor in the development of diabetic cardiomyopathy, comprising functional and structural abnormalities in the heart, including diastolic and/or systolic dysfunction, altered cardiac contractility, cell hypertrophy, apoptosis and interstitial fibrosis.

## Materials and Methods

### Reagents and Chemicals

Poly(D,L-lactide-co-glycolide; lactide:glycolide molar ratio *50:50*, MW *30,000–60,000*), PVA (87–89% hydrolyzed, MW 13,000–23,000), Rh B (MW 479.01), and mannitol were purchased from Sigma-Aldrich. DCM was purchased from VTC. All other chemicals and solvents were of HPLC grade. The primary rat cardiac myocytes were purchased from Lonza and grown as described by the manufacturer protocol (CM561-6 03/13). In brief cell were grown in Rat Cardiac Myocyte Growth Media containing Horse Serum, Fetal Bovine Serum [FBS], and Gentamicin/Amphotericin-B [GA] and incubated at 37°C in a 5% CO_2_ incubator. Before the experiments cells were serum deprived for 12 h then treated with HG, mannitol or the different concentration of the RhoB-loaded PLGA NPs.

### Preparation of Rhodamine-Loaded Nanoparticles

Nanoparticles loaded with Rh B were fabricated by a single emulsion-solvent evaporation technique (Scholes et al., 1993). Briefly, a solution of 50 mg of PLGA in 1 mL of DCM containing 0.01 mg/mL Rh B, was mixed with 10 mL of 2.5% PVA aqueous solution. This mixture was sonicated using a probe sonicator (Qsonica) set at 20 W for 10 min to produce the oil-in-water emulsion. The organic phase was evaporated during 45 min using a rotary evaporator under partial vacuum. The NPs were recovered by ultracentrifugation at 72,000.00 ×*g* for 10 min and washed three times with deionized water in order to remove free and surface adsorbed Rh B. The washing solutions were eliminated by centrifugation as described previously. The purified NPs were lyophilized. The supernatant removed in the first step and the washing solutions were combined together and used to measure the amount of non-entrapped Rh B by spectrophotometric analysis.

### Nanoparticles Characterization

Particles size measurements and distribution were determined by DLS analyzer (DLS/NanoBrook 90 Plus Particle Size Analyzer – Brookhaven) at 25°C. The PLGA NPs were dispersed in double distilled water and analyzed in triplicates with three readings per nanoparticle sample. The polydispersity was calculated based on the volumetric distribution of particles. The NPs zeta potential was measured by DLS (Zetasizer Malvern ZPS) at 25°C.

The NPs morphology and size were observed by scanning electron microscope (Mira Tescan) operated at 30 kV of beam energy. A drop of the sample was deposited and spread at the center of the carbon tape. After drying, the sample was sputter coated with 2 nm gold.

### Determination of Rhodamine B Encapsulation Efficiency

The amount of Rh B trapped in the NPs was determined by subtracting the amount of Rh B, present in the supernatant of the nanoparticle suspension removed after centrifugation combined to the supernatants collected during the cycles of nanoparticle washes, from the initial quantity of Rh B used for nanoparticle preparation. The Rh B in the supernatant was measured using fluorescence spectrometry (λexcitation = 553 nm, λemission = 574 nm). The non-encapsulated Rh B concentration was determined using a calibration curve. The EE was determined as follow:

A⁢m⁢o⁢u⁢n⁢t⁢  o⁢f⁢  e⁢n⁢c⁢a⁢p⁢s⁢u⁢l⁢a⁢t⁢e⁢d⁢ R⁢h⁢  B=  I⁢n⁢i⁢t⁢i⁢a⁢l⁢ R⁢h⁢ B⁢ a⁢m⁢o⁢u⁢n⁢t⁢ u⁢s⁢e⁢d⁢ f⁢o⁢r⁢  t⁢h⁢e⁢ p⁢r⁢e⁢p⁢a⁢r⁢a⁢t⁢i⁢o⁢n−A⁢m⁢o⁢u⁢n⁢t⁢ o⁢f⁢ R⁢h⁢ B⁢ i⁢n⁢ t⁢h⁢e⁢ c⁢o⁢m⁢b⁢i⁢n⁢e⁢d⁢ supe⁢r⁢n⁢a⁢tant⁢s

$$E\,n\,c\,a\,p\,s\,u\,l\,a\,t\,i\,o\,n\,\;e\,f\,f\,i\,c\,i\,e\,n\,c\,y\,\mathrm{(\%) =}\frac{A\,m\,o\,u\,n\,t\,\;o\,f\,\;e\,n\,c\,a\,p\,s\,u\,l\,a\,t\,e\,d\,\;R\,h\,\;B}{I\,n\,i\,t\,i\,a\,l\,\;R\,h\,\;B\,\;a\,m\,o\,u\,n\,t\,\;u\,s\,e\,d\,\;f\,o\,r\,\;t\,h\,e\,\;p\,r\,e\,p\,a\,r\,a\,t\,i\,o\,n}\times 100$$

### *In vitro* Release

The dialysis diffusion technique was used to evaluate Rh B release from PLGA NPs. Briefly, 2.5 mg of the lyophilized PLGA NPs were suspended in 500 μl PBS solution (PBS, 0.01 M) “inner phase” and poured in a dialysis bag (molecular weight cut-off: 1,000 Da). The dialysis bag was emerged into 35 ml PBS buffer “outer phase” with continuous stirring and was kept at 37°C. Four hundred microliter samples were pipetted from the outer phase at different time intervals and were replaced with same volume of fresh PBS. The experiments were performed in triplicate at pH 7.2. The amount of Rh B released was quantified using high performance liquid chromatography with fluorescence detector (λexcitation = 539 nm, λemission = 573 nm).

### Studies of Nanoparticles- Cardiac Myocytes Interaction *In vitro*

*In vitro* experiments were performed to elucidate the interaction of PLGA NPs with cardiac myocytes.

The cytotoxicity assessment of the RhoB-loaded PLGA NPs was performed using the MTT assay (Gomez et al., 1997; Fajardo et al., 2006). Approximately 1 × 10^5^ cells/mL of cardiomyocytes in their exponential growth phase were seeded in a flat-bottomed 96-well polystyrene coated plate and were incubated for 24 h at 37°C in a 5% CO_2_ incubator. Different concentrations of NPs were added to the plate. HG, known to induce cardiomyotoxicity was used as a positive control (Kobayashi et al., 2012) and mannitol, as a negative control. After 20 and 44 h of incubation, 10 μL of MTT reagent was added to each well and was further incubated for 4 h. Formazan crystals formed after 4 h in each well were dissolved in 150 μL of detergent and the plates were read immediately in a microplate reader at 570 nm. Wells with complete medium, NPs and MTT reagent, without cells were used as blanks.

Cellular apoptosis was assessed using the cellular DNA fragmentation test on cultured cardiomyocytes treated with different concentration of the RhoB-loaded PLGA NPs. HG, known to induce apoptosis, was used as a positive control and mannitol was added to the experiments to serve as a negative control. The test was performed using a commercial ELISA that detects 178 BrdU-labeled DNA fragments according to the manufacturer protocol (Roche Diagnostics).

The kinetic cellular uptake of RhB-loaded PLGA NPs was assessed by confocal laser scanning microscopy (CLSM, Zeis LSM 710). Cells were grown on coverslips in a 6-well tissue culture plate at a concentration of 5 × 10^4^ cells/well. Cells were incubated at 37°C under 5% CO_2_ and were maintained in a rat cardiac myocyte growth medium supplied by the manufacturer as described in the material and method section. Cells were incubated with RhB-loaded PLGA NPs at a concentration of 200 ng/mL for 1, 6, 12, and 24 h, respectively. Afterward, they were washed with PBS and fixed with 5% paraformaldehyde in PBS. Canadian Balsam was dropped on the slides to seal the cell samples after PBS washes. DAPI was used to stain the cells’ nuclei in blue. Quantification of cellular uptake was based on a modified method previously described by Blechinger et al. (2013). In brief, and in order to determine the number of particles forming each object the total intensity of particles or agglomerates is measured and compared to the intensity of a single particle using image J.

### Statistical Analysis

Results are expressed as means ± standard errors (SE). Statistical significance was assessed by the Student’s *t*-test with Prism 6 software (GraphPad Software). Significance was determined as a probability (*P* value) of less than 0.05.

## Results And Discussion

### Optimization of the PLGA Nanoparticles Parameters

Experimental parameters such as the sonication time, PVA concentration and organic to aqueous phase ratio were investigated for particle size, PDI and EE. The results are summarized in **Table 1**. All the experiments were conducted by varying one parameter and keeping all the other processing parameters constant.

**Table 1:** Comparison of particle size, PDI and EE of Rho-loaded PLGA NPs prepared by varying different parameters.

Sonication time (min)	Mean diameter (nm)	PDI	EE (% ± SD)
**Influence of sonication time**			
3	204 ± 12	0.18 ± 0.01	17 ± 10.41
5	190 ± 12	0.19 ± 0.03	25 ± 12.01
10	184 ± 7	0.19 ± 0.01	40 ± 2.94

**PVA content (% w/v)**	**Mean diameter (nm)**	**PDI**	**EE (% ± SD)**

**Influence of PVA content**			
0.5	229 ± 17	0.22 ± 0.03	72 ± 4.58
1	222 ± 5	0.23 ± 0.02	65.5 ± 1.91
2.5	184 ± 7	0.19 ± 0.01	40 ± 2.94

**org/aq**	**Mean diameter (nm)**	**PDI**	**EE (% ± SD)**

**Influence of organic to aqueous phase volume**			
0.5:10	232 ± 12	0.24 ± 0.01	50.25 ± 1.71
1:10	184 ± 7	0.19 ± 0.01	40 ± 2.94
2:10	195 ± 7	0.23 ± 0.01	34.5 ± 2.52

*PDI, polydispersity index; EE, encapsulation efficiency. Values are presented as mean ± standard deviation, n = 3.*

The external energy applied via sonication is a crucial step in the emulsification process. It will allow the breakdown of the emulsion causing a decrease in the mean diameter of NPs. An increase in the sonication time from 3 to 10 min leads to a decrease in the mean diameter of NPs from 204 to 184 nm. Sonication caused no considerable change in the PDI. The EE exhibited a slight upward trend with increasing sonication. Thus, a stable formulation was achieved after 10 min sonication with an average diameter of 184 nm.

The PVA is a common stabilizer used to prevent the aggregation of PLGA NPs. The effect of PVA on nanoparticle size has been somewhat controversial. In some papers, the diameter of the PLGA NPs was found to decrease with the increase of the PVA concentration (Lee et al., 1999; Mainardes and Evangelista, 2005; Budhian et al., 2007) in others an opposite trend was observed (Agrahari et al., 2009). Some researchers reported that as the PVA concentration is increased, the mean diameter of NPs first decreases and then gradually increases (Budhian et al., 2007). In our case, we observed that the mean size of PLGA NPs decreased with increasing PVA concentration from 0.5 to 2.5% (w/v). The surfactant exerts its stabilizing effect by adsorbing at the NPs interface, thus reducing the surface tension between the two phases, preventing the aggregation of NPs and thereby lowering their size. It is well known that PVA remains associated at the surface of the polymer and is difficult to remove even after successive washing. Sahoo et al. (2002) reported that NPs with higher amount of residual PVA has relatively lower cellular uptake. For instance, a 2.5% w/v was adopted and no attempts were made to increase further the PVA concentration. The PDI remained unchanged while passing from 0.5 to 1% PVA then decreased with the increase of PVA to 2.5%. It was also observed that an increase of the PVA concentration is accompanied by a decrease in the EE.

The organic to aqueous phase ratio is another factor that can impact the evaporation phase and the NPs formation process. The size of the NPs exhibited an initial decrease from 232 to 184 nm when the concentration of the organic phase increased from 4.76 to 9.09% by changing the ratio of the organic to aqueous phase from 0.5:10 to 1:10 (v/v), respectively. The increase in the organic phase volume prevents the coalescence of NPs and lead to the decrease in the mean diameter. On further increasing the concentration from 9.09 to 16.67%, a slight increase in the particle size from 184 to 195 nm was observed. This finding may be explained by the increase in the viscosity of the emulsion formed implying a lower net shear stress, therefore leading to the formation of larger size NPs. An optimum ratio of 1:10 was selected. The polydispersity decreased then increased by changing the concentration of the organic phase from 4.76 to 9.09% and 16.675, respectively. The EE decreased as the volume of the organic phase increases from 4.76 to 16.67%. This can be explained by the fact that increasing the solvent volume increases the time of solvent evaporation allowing more time for drug diffusion and decreases the drug content.

The zeta potential is another important parameter considered as a key indicator for the stability of nanoparticle in water suspension. The zeta potential of the optimized RhB-loaded PLGA NPs was -21.7 ± 5.64 mV. Thus, the repulsion among the negatively charged NPs provides stability and prevents aggregation (Honary and Zahir, 2013).

**FIGURE 1:**
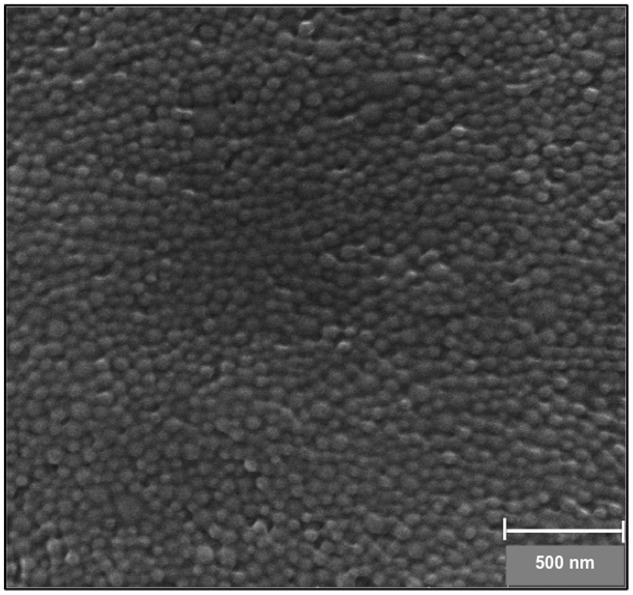
**Scanning electron micrograph of RhB-loaded PLGA NPs**.

The morphological properties and the size of the NPs were characterized by SEM. PLGA NPs were found to have an average diameter of 84.31 ± 9.31 nm with a smooth and spherical surface morphology (**Figure 1**). There is often a discrepancy between DLS and SEM measurements that is attributed to the hydrodynamic radius calculated via DLS and the estimated of projected area diameter obtained using SEM.

### *In vitro* Release

**FIGURE 2:**
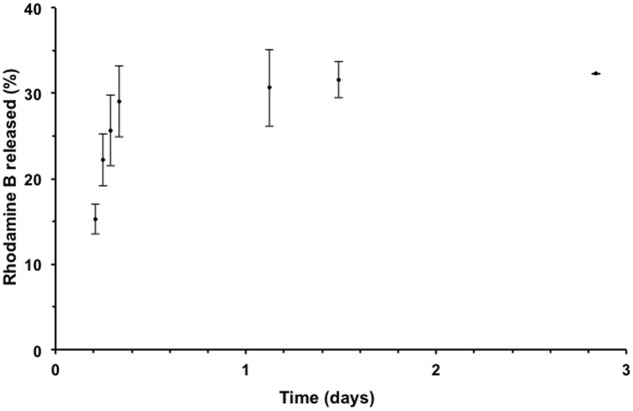
**Release profile of Rh B from PLGA NPs in phosphate buffered saline (PBS) solution at 37°C using dialysis method.** Data points are presented as mean ± standard deviation, *n* = 3.

The release profile of RhB-loaded PLGA NPs was measured at pH 7.4 and found to follow a biphasic pattern; a burst release followed by a slower release rate. As shown in **Figure 2**, the release profile was recorded over a 3-day period. In the first stage, 29% of the Rh B was released within the first 8 h. This initial burst release may be attributed to the Rh B molecules adsorbed or encapsulated close to the surface (Magenheim et al., 1993; Budhian et al., 2008). The slower second phase is related to the entrapped Rh B inside the polymeric NPs. Analogous release profile was observed previously for PLGA NPs (Cartiera et al., 2009; Abulateefeh et al., 2013).

### Cytotoxicity and Confocal Microscopy

**FIGURE 3:**
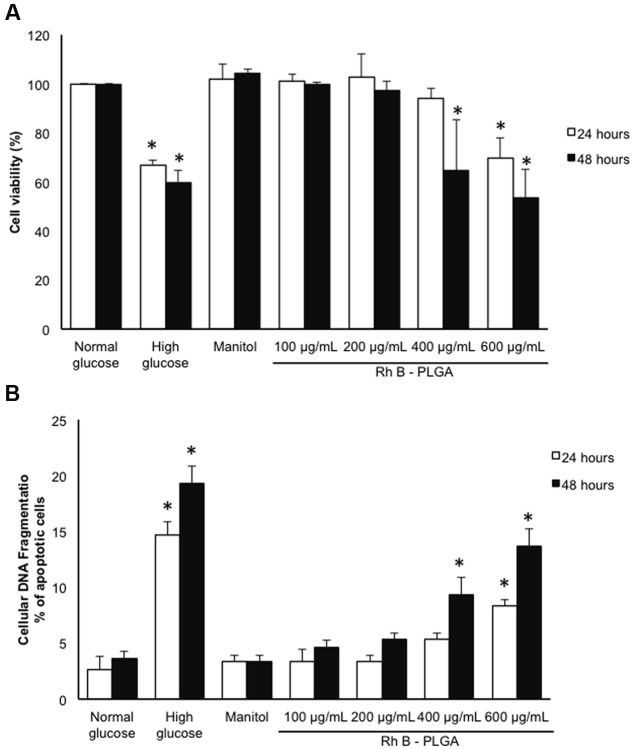
**(A)** Cytotoxicity analyses results by MTT assay after 24 and 48 h incubation with RhB-loaded PLGA NPs. **(B)** % of cardiomyocyte apoptosis after 24 and 48 h incubation with RhB-loaded PLGA NPs as assessed by the cellular DNA fragmentation assay. Values are the mean ± standard deviation from three independent experiments. ^∗^*P* < 0.05 vs. normal glucose.

The cytotoxicity profile of the NPs was conducted using the MTT assay. Only cells that are viable after 24 or 48 h exposed to different concentration of NPs were capable of metabolizing a dye [3-(4,5-dimethylthiozol-2-yl)-2,5-diphenyl tetrazolium bromide] efficiently and the purple colored precipitate which is dissolved in a detergent was analyzed spectrophotometrically. Our results show that cardiomyocyte cultured cells exposed to 20 mM HG for 24 and 48 h undergo cell death assessed by the decrease in the cellular viability assay. Mannitol, an osmotic control, and as expected (Eid et al., 2009, 2013) did not show any effect on cardiomyocyte survival/toxicity ruling out an osmotic effect. The same profile of cardiomyocyte survival was obtained by assessing cellular apoptosis using the cellular DNA fragmentation assay. These results were corroborated with previously published data (Duffy et al., 2006). Importantly our results show that no significant cytotoxic or apoptotic effects were observed after 24 h of exposure to 100, 200, and 400 μg/mL of RhB-loaded PLGA NPs. Cells treated with the highest concentration of NPs showed significantly low viability when compared to cells treated with mannitol. On the other hand, 48 h of exposure to 400 and 600 μg/mL led to a significant decrease in cardiomyocyte viability and an increase in cellular apoptosis (*p* < 0.05). Thus, it can be concluded that cell viability decreases when exposure time increases and that concentrations up to 200 μg/mL do not possess any significant cytotoxic or apoptotic effect after 24 and 48 h of exposure (**Figures 3A,B**).

**FIGURE 4:**
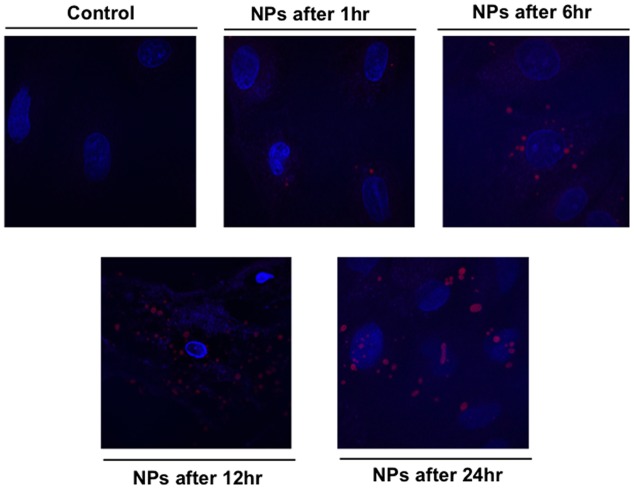
**Confocal fluorescence image showing cardiac myocytes exposed to 200 ng/mL RhB-loaded PLGA NPs over different time period.** The blue color presents the nucleus stained with DAPI.

**FIGURE 5:**
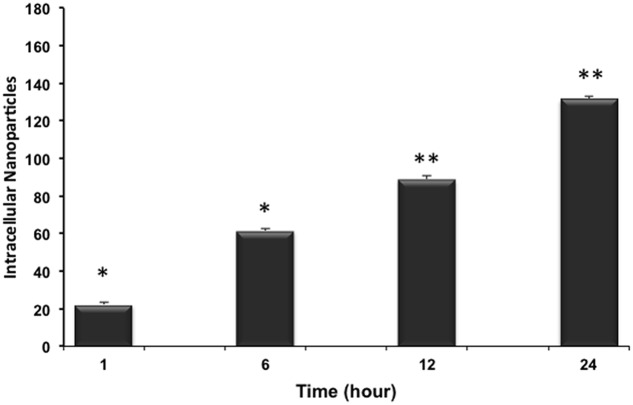
**Uptake kinetics of RhB-loaded PLGA NPs.** Within the 24 h the mean number of internalized NPs in cardiomyocytes increases significantly and almost linearly. Results are statistically different (^∗^*p* < 0.05; ^∗∗^*p* < 0.01) for time points 1–24 h. Results are expressed as mean ± standard deviation, *n* = 3.

The kinetic uptake of RhoB-loaded PLGA NPs is a crucial factor to study the interaction between NPs and cells. **Figure 4** shows representative images of cardiac myocytes exposed to 200 ng/mL RhB-loaded PLGA NPs at different incubation time points of 1, 6, 12, and 24 h. Quantification is based on a modified method previously described (Blechinger et al., 2013). In brief, and in order to determine the number of particles forming each object the total intensity of particles or agglomerates is measured and compared to the intensity of a single particle using image J. **Figure 4** clearly demonstrates that NPs (red) are localized within the cellular boundaries showing their uptake into the cytoplasm. The NPs were distributed more or less regularly throughout the cell and the cellular uptake appeared to increase with exposure time from 1 to 24 h. Around 20 cardiac cells were analyzed per time point applying the same controlled conditions. The average fluorescence intensities at each time point are presented in **Figure 5**. In fact, these results are in line with what was described by Cartiera et al. (2009) whereby showed that the rate and extent of PLGA NPs uptake among cell lines differs, most likely due to regulatory mechanisms inherent to the particular cell type.

## Conclusion

In this present work, PLGA NPs loaded with the fluorescent agent Rh B were prepared using single emulsion solvent evaporation technique. Spherical particles with a diameter of 184 nm and 40% EE were obtained. Release studies revealed that 29% of Rh B was release within the first 8 h. These NPs were designed as models to study their interactions with cardiac myocytes. No significant *in vitro* cytotoxic effect was observed in cardiac myocytes after 24 and 48 h of exposure to 100 and 200 μg/mL consecutively. Cells exposed to 400 μg/mL of RhB-loaded PLGA NPs showed decrease in cell viability after 48 h of exposure, an effect not seen after 24 h of exposure at the same concentration. Furthermore, the confocal images demonstrate that PLGA NPs are taken up intracellularly. These findings, all together, make these NPs good candidate for future targeted siRNA delivery for cardiac myocytes, making it possible to deliver drugs, inhibitors and other therapeutic proteins to local areas of disease to maximize clinical benefit while limiting unwanted side effects.

## Author Contributions

AJ performed experiments; RM conceived the project, designed experiments, analyzed data, and wrote the manuscript. All authors read and approved the final manuscript.

## Abbreviations

DAPI: 4'6-diamidino-2-phenylindole
DCM: dichloromethane
DLS: dynamic light scattering
DMEM: Dulbecco’s Modified Eagle Medium
DPBS: Dulbecco’s phosphate buffer saline
EE: encapsulation efficiency
HG: high glucose
NPs: nanoparticles
PBS: phosphate buffer saline
PDI: polydispersity index
PGA: poly (glycolic acid)
PLA: poly(lactic acid)
PLGA: poly(D,L-lactide-co-glycolide)
PVA: poly (vinyl alcohol)
Rh B: rhodamine B
SEM: scanning electron microscopy.
